# Changes in Corneal Biomechanics and Wavefront Following Eyelid Surgery

**DOI:** 10.7759/cureus.113381

**Published:** 2026-07-25

**Authors:** Khushdeep Abhaypal, Parul Chawla Gupta, Manpreet Singh, Manu Saini, Padma Thinlass, Pankaj Gupta

**Affiliations:** 1 Ophthalmology, Postgraduate Institute of Medical Education and Research, Chandigarh, Chandigarh, IND; 2 Computational Drug and Vaccine Discovery Laboratory, Faculty of Applied Sciences and Biotechnology, Shoolini University, Solan, IND

**Keywords:** corneal biomechanics, eyelid mass, ‎eyelid surgery, ocular response analyser, wavefront aberrations

## Abstract

Introduction: This study evaluated corneal biomechanics and wavefront aberrations in patients with eyelid masses and documented changes in these parameters following surgical excision in a Northwest Indian population through a prospective interventional study.

Methods: Twenty eyes of 20 patients requiring surgical excision of eyelid masses were enrolled. Preoperative and postoperative assessments included a comprehensive ocular examination, corneal biomechanics evaluation using an ocular response analyzer, and wavefront aberrometry with the I-Trace system.

Results: Corneal biomechanics were assessed preoperatively and postoperatively in all patients. No statistically significant changes were observed in corneal hysteresis or corneal resistance factor following surgery, regardless of lesion location in the upper (P=0.458) or lower eyelids (P=0.154). Similarly, wavefront aberrations, including lower-order aberrations (LOAs), higher-order aberrations, spherical aberrations (SAs), coma, and trefoil, showed no significant postoperative differences overall. However, patients with lesions ≥5 mm in size exhibited significant changes in astigmatism, SAs (P=0.008), coma (P=0.050), and total LOAs (P=0.049) postoperatively. Patients with lesions ≥5 mm demonstrated significant postoperative changes in selected corneal wavefront parameters, whereas no significant changes were observed in corneal biomechanical parameters.

Conclusion: Surgical excision of larger eyelid masses (≥5 mm) is associated with significant postoperative changes in corneal wavefront parameters, particularly in astigmatism, SAs, coma, and LOAs, while corneal biomechanics remain stable. This highlights the role of lesion size in influencing corneal wavefront changes, warranting further investigation in larger cohorts.

## Introduction

Eyelids play a vital role in ocular health and facial aesthetics, with their position significantly influencing corneal contour and biomechanics. Alterations in eyelid position, pathologies such as chalazia, or prolonged downgaze can impact corneal topography and induce astigmatism [1-6]. Corneal hysteresis (CH), a biomechanical property measurable with an ocular response analyzer (ORA), can be affected by long-standing eyelid lesions or malpositions [7,8]. Similarly, eyelid pathologies, including masses or malpositions, can disrupt tear film dynamics, altering the corneal wavefront and affecting contrast sensitivity and overall visual quality, measurable through wavefront aberrometry [9].

Studies have shown that eyelid chalazia can significantly increase higher-order aberrations (HOAs), astigmatism, and corneal wavefront changes, with larger and upper eyelid chalazia causing the greatest impact [10-12]. Previous studies have also demonstrated that upper and lower eyelid surgeries can induce transient corneal topographic and refractive changes, with mean dioptric shifts ranging from 0.19 D to 0.60 D. These alterations, including HOAs, highlight the influence of eyelid pressure on corneal biomechanics and wavefront [13-15].

The primary objective of this prospective interventional study was to evaluate postoperative changes in corneal biomechanics, specifically CH and corneal resistance factor (CRF), following surgical excision of eyelid masses. The secondary objectives were to evaluate postoperative changes in corneal wavefront parameters, including lower-order aberrations (LOAs), HOAs, spherical aberrations (SAs), coma, trefoil, keratometry, and pachymetry, and to assess the influence of lesion size and location on these changes. We hypothesized that surgical excision of eyelid masses would not significantly alter corneal biomechanics, whereas postoperative changes in corneal wavefront parameters may occur depending on lesion characteristics.

## Materials and methods

This prospective interventional study was conducted at Advanced Eye Centre, Postgraduate Institute of Medical Education and Research (PGIMER), Chandigarh, a tertiary referral centre in Northwest India, over a study period from January 2020 to December 2021. The study adhered to the tenets of the Declaration of Helsinki and was approved by the Institutional Ethics Committee. Written informed consent was obtained from all participants prior to enrollment.

Patients aged ≥18 years presenting to the ophthalmology outpatient department with eyelid masses requiring surgical excision were consecutively recruited. Inclusion criteria comprised patients with a clear cornea, no prior ocular surgery, and no history of ocular trauma. Exclusion criteria included glaucoma, ocular hypertension, degenerative myopia, clinically significant dry eye disease, and unwillingness to participate.

All participants underwent a comprehensive ophthalmic evaluation, including visual acuity assessment, refraction, slit-lamp examination, and posterior segment evaluation. Corneal biomechanics were assessed using an ORA (Reichert Ophthalmic Instruments, Buffalo, NY, USA), which measured CH, CRF, corneal-compensated intraocular pressure (IOPcc), and Goldmann-correlated intraocular pressure (IOPg). Corneal wavefront analysis was performed using the iTrace aberrometer (Tracey Technologies, Houston, TX, USA), evaluating LOAs, HOAs, SAs, coma, trefoil, keratometry (Kmax, Kmean), and pachymetry. All preoperative and postoperative assessments were performed using the same instruments and standardized measurement protocols under similar examination conditions to minimize measurement variability.

Detailed documentation of eyelid lesions included size (in mm), location (upper/lower eyelid; medial, middle, or lateral one-third), laterality, vascularity, overlying skin condition, eyelid position, palpebral aperture dimensions, and eyelid mobility.

All patients underwent surgical excision under aseptic conditions following standard ophthalmic surgical protocols. Depending on the type and location of the lesion, procedures included incision and curettage (for chalazion) or complete excision biopsy (for other benign eyelid masses). Local anesthesia (2% lignocaine with or without adrenaline) was administered. For chalazion, a conjunctival approach with vertical incision and curettage of granulomatous material was performed, while for other lesions, an external approach with careful dissection and complete excision was undertaken. All surgical procedures were performed by a single experienced oculoplastic surgeon using standardized surgical techniques appropriate for the lesion type. Hemostasis was achieved, and closure was performed when required. All excised eyelid masses were routinely submitted for histopathological examination as part of the institutional protocol to confirm the clinical diagnosis. Histopathological findings were not included as study outcome measures. Postoperative care included topical antibiotics and anti-inflammatory medications. Topical corticosteroids were not routinely prescribed.

Patients were reviewed at six weeks postoperatively, at which time all baseline investigations were repeated, including refraction, ORA measurements, and wavefront aberrometry. 

The primary outcome was the change in corneal biomechanics following surgical excision of eyelid masses, assessed by CH and CRF.

Secondary outcomes included postoperative changes in corneal wavefront parameters (lower-order aberrations, higher-order aberrations, SAs, coma, trefoil), keratometry (Kmax and Kmean), pachymetry, and subgroup analyses evaluating the influence of lesion size and lesion location on these parameters.

A formal sample size calculation was not performed due to the exploratory nature of the study and limited prior data on combined corneal biomechanics and wavefront changes in eyelid masses. A convenience sample of 20 eyes of 20 patients was included over the study period, similar to previous prospective interventional studies evaluating corneal changes following eyelid pathology or surgery. This sample size was considered adequate to detect clinically meaningful changes and to generate preliminary data for future larger studies.

Data analysis was performed using IBM SPSS Statistics for Windows, Version 27 (Released 2019; IBM Corp., Armonk, New York, United States). Normality was assessed using the Shapiro-Wilk test. Quantitative variables were expressed as mean ± standard deviation. A paired-samples t-test was used to compare preoperative and postoperative values. One-way analysis of variance (ANOVA) was used for subgroup comparisons. A P-value < 0.05 was considered statistically significant.

## Results

The mean age of the study participants was 35 ± 16 years (range: 19-70 years). The cohort comprised 12 female participants (60%) and eight male participants (40%), with a male-to-female ratio of 0.6:1. The mean logMAR visual acuity at presentation was 0.15, and the mean duration of symptoms prior to presentation was 9.5 months.

Lesion size exceeded 5 mm in 12 patients (60%), with a mean size of 6.1 ± 1 mm in this subset (n = 11). In the remaining subset of patients (n = nine), the mean lesion size was 3.7 ± 0.6 mm.

The mean preoperative and postoperative logMAR visual acuity across all participants was 0.15 ± 0.08 and 0.11 ± 0.03, respectively, with no statistically significant improvement noted postoperatively (paired t-test, P = 0.317). Postoperative visual acuity according to the lesion site was 0.11 ± 0.03, 0.13 ± 0.05, and 0.11 ± 0.03 logMAR units for lesions located in the middle one-third, medial one-third, and lateral one-third of the eyelid, respectively, with no statistically significant differences among these groups (ANOVA, P = 0.665).

Corneal biomechanics

Corneal biomechanics were evaluated preoperatively and postoperatively in all participants. The mean preoperative corneal-compensated intraocular pressure (IOPcc) was 15.6 mmHg. Postoperative changes in corneal biomechanical parameters, including CRF, were not statistically significant at six weeks following surgery. These findings are summarized in Table 1. 

**Table 1:** Corneal biomechanics comparison IOP: Intraocular pressure. Values are expressed as mean ± standard deviation. Preoperative and postoperative comparisons were performed using the paired-samples t-test; corresponding t-statistics and P-values are presented.

Parameter	Preoperative (Mean±SD)	Postoperative (Mean ±SD)	t (df =19)	P-value Paired t-test
Corneal compensated IOP	15.6±3.6	14.8±3.8	t = 1.34	0.198
Corneal resistance factor	9.2±1.6	9.4±1.3	t = -0.40	0.695
Corneal hysteresis	9.6±1.5	9.9±1.6	t = -1.20	0.224
Goldman compensated IOP	13.8±3.5	13.1±3.2	t = 0.94	0.358

Preoperative and postoperative corneal wavefront parameters, including LOAs, HOAs, SAs, coma, and trefoil, were compared. No statistically significant postoperative changes were observed in these parameters in the overall study population. The detailed wavefront and pachymetry parameters are summarized in Table 2.

**Table 2:** Preoperative and postoperative corneal wavefront (n = 20) LO: Lower order; HO: higher order. Values are expressed as mean ± standard deviation. Preoperative and postoperative comparisons were performed using the paired-samples t-test; corresponding t-statistics and p-values are presented.

Parameter	Preoperative (Mean±SD)	Postoperative (Mean ±SD)	t (df=19)	P-value Paired t-test
K_max_	45±3.2	44.8±2.1	t = 0.39	0.699
K_mean_	43.4±1.5	43.6±1.6	t = -1.08	0.289
Pachymetry				
Centre	518±30.6	517±29.5	t = 0.43	0.673
Apex	519±30.5	518±29.9	t = 0.54	0.595
Thinnest point	512±27	515±29.7	t = -0.74	0.472
Astigmatism	0.32±0.2	0.35±0.25	t = -0.69	0.497
LO (total)	0.33±0.2	0.37±0.25	t = -0.67	0.510
HO (total)	0.23±0.13	0.24±0.15	t = -0.13	0.894
Spherical aberration	0.10±0.05	0.11±0.07	t = -0.02	0.984
Coma	0.01±0.06	0.11±0.07	t = -0.60	0.554
Trefoil	0.10±0.07	0.12±0.10	t = -0.83	0.415

Comparison based on the lesion size

Lesions were compared based on their size (<5 mm vs ≥5 mm) in both the upper and lower eyelids. Parameters analyzed included CRF, CH, lower- and higher-order aberrations, SAs, coma, trefoil, and astigmatism. Preoperative and postoperative comparisons of these parameters were also performed.

For lesions ≥5 mm, postoperative changes in lower-order aberrations were statistically significant in the lower eyelids (ANOVA, P = 0.049) but not in the upper eyelids (ANOVA, P = 0.727). SAs also demonstrated significant postoperative changes in the lower eyelids (ANOVA, P = 0.008) but not in the upper eyelids (ANOVA, P = 0.599). Similarly, coma showed significant postoperative changes in the lower eyelids (ANOVA, P = 0.050) but not in the upper eyelids (ANOVA, P = 0.410). The detailed data are summarized in Table 3.

**Table 3:** Comparison based on the lesion size CRF: Corneal resistance factor; CH: corneal hysteresis; LO: lower order; HO: higher order. Values are expressed as mean ± standard deviation. Comparisons were performed using one-way analysis of variance (ANOVA); corresponding F-statistics and p-values are reported.

Size/site	Less than 5 mm		More than 5 mm	
	Preoperative	Postoperative	Preoperative	Postoperative
CRF				
Upper eyelid	8.2±1.5	8.9±1	9.1±1.6	9.3±0.7
Lower eyelid	9.2±1.6	9.1±1.6	10.2±1.4	10±1.9
P-value	0.372	0.769	0.263	0.452
F-value	0.64	0.11	1.28	0.52
CH				
Upper eyelid	9.3±1.2	10±0.9	9.2±1.6	9.5±1.5
Lower eyelid	9.3±0.9	9.8±1.8	10.6±2.1	10.5±2.2
P-value	0.995	0.812	0.259	0.412
F-value	0.03	0.07	1.39	0.68
LO aberrations				
Upper eyelid	0.42±0.2	0.46±0.2	0.25±0.21	0.26±0.18
Lower eyelid	0.43±0.2	0.39±0.3	0.24±0.14	0.38±0.27
P-value	0.944	0.727	0.831	0.049
F-value	0.01	0.14	0.05	4.12
HO aberrations				
Upper eyelid	0.29±0.22	0.25±0.13	0.22±0.13	0.20±0.15
Lower eyelid	0.20±0.08	0.21±0.08	0.24±0.10	0.30±0.23
P-value	0.436	0.611	0.714	0.730
F-value	0.58	0.26	0.19	0.11
Spherical aberrations				
Upper eyelid	0.16±0.19	0.13±0.03	0.06±0.05	0.06±0.03
Lower eyelid	0.09±0.06	0.10±0.10	0.11±0.07	0.14±0.03
P-value	0.475	0.599	0.248	0.008
F-value	0.48	0.31	1.36	7.92
Coma				
Upper eyelid	0.14±0.10	0.09±0.06	0.08±0.04	0.08±0.03
Lower eyelid	0.12±0.04	0.12±0.03	0.07±0.03	0.15±0.13
P-value	0.693	0.410	0.799	0.050
F-value	0.18	0.66	0.09	3.75
Trefoil				
Upper eyelid	0.14±0.10	0.15±0.09	0.09±0.03	0.08±0.03
Lower eyelid	0.09±0.08	0.09±0.05	0.09±0.07	0.19±0.18
P-value	0.504	0.321	0.955	0.177
F-value	0.42	0.98	0.02	1.81
Astigmatism				
Upper eyelid	0.42±0.24	0.46±0.25	0.22±0.22	0.21±0.15
Lower eyelid	0.43±0.20	0.39±0.21	0.23±0.11	0.38±0.27
P-value	0.944	0.727	0.925	0.029
F-value	0.01	0.12	0.02	4.88

Comparison based on the site of lesions

Lesions were compared based on their location on the upper or lower eyelids and their site (middle, medial, or lateral one-third of the eyelid). Parameters analyzed included CRF, CH, lower- and higher-order aberrations, SAs, coma, and trefoil.

No statistically significant differences were observed between the groups. Additionally, comparison of these parameters preoperatively and at six weeks postoperatively also revealed no statistically significant changes. The detailed data for these comparisons are summarized in Table 4.

**Table 4:** Comparison based on the site of lesions on the eyelid CRF: Corneal resistance factor; CH: corneal hysteresis; LO: lower order; HO: higher order. Comparisons were performed using one-way analysis of variance (ANOVA); corresponding F-statistics and p-values are reported.

Site	Upper Eyelid		Lower Eyelid	
	Preoperative	Postoperative	Preoperative	Postoperative
CRF				
Middle one third	9±1.8	9.2±0.6	11±1.2	11.1±1.1
Medial one third	8.1±0	10.1±0	9±0.1	8.3±1.6
Lateral on third	8.6±1.6	8.8±1	9.2±1.6	9.1±1.6
P-value	0.870	0.458	0.207	0.145
F-value	0.21	0.88	1.74	2.02
CH				
Middle one third	8.8±1.3	9.3±1.4	11.4±2.1	11.4±0.6
Medial one third	8.4±0	9.8±0	9.3±1.9	9±3.4
Lateral on third	9.9±1.5	10.3±1.22	9.3±0.9	9.8±1.8
P-value	0.485	0.571	0.193	0.385
F-value	0.74	0.61	1.91	1.02
LO aberrations				
Middle one third	0.2±0.27	0.23±0.2	0.24±0.16	0.28±0.17
Medial one third	0.49±0	0.45±0	0.31±0.15	0.53±0.4
Lateral on third	0.37±0.2	0.45±0.26	0.43±0.20	0.4±0.31
P-value	0.566	0.351	0.410	0.681
F-value	0.63	1.09	0.97	0.41
HO aberrations				
Middle one third	0.31±0.2	0.23±0.16	0.17±0.04	0.18±0.04
Medial one third	0.13±0	0.18±0	0.35±0.04	0.47±0.34
Lateral on third	0.2±0.07	0.22±0.16	0.20±0.08	0.21±0.08
P-value	0.535	0.951	0.063	0.133
F-value	0.71	0.08	3.10	2.19
Spherical aberrations				
Middle one third	0.11±0.2	0.07±0.04	0.11±0.04	0.14±0.03
Medial one third	0.06±0	0.11±0	0.11±0.14	0.14±0.05
Lateral on third	0.09±0.04	0.10±0.05	0.09±0.06	0.10±0.10
P-value	0.945	0.488	0.932	0.763
F-value	0.09	0.69	0.11	0.29
Coma				
Middle one third	0.11±0.09	0.10±0.03	0.08±0.24	0.07±0.02
Medial one third	0.09±0	0.02±0	0.05±0.05	0.26±0.18
Lateral on third	0.09±0.05	0.09±0.05	0.12±0.04	0.12±0.03
P-value	0.900	0.310	0.185	0.078
F-value	0.13	1.22	1.78	2.84
Trefoil				
Middle one third	0.12±0.08	0.09±0.03	0.08±0.04	0.10±0.02
Medial one third	0.03±0	0.10±0	0.11±0.12	0.32±0.26
Lateral on third	0.11±0.05	0.13±0.11	0.09±0.08	0.09±0.06
P-value	0.486	0.844	0.941	0.099
F-value	0.47	0.99	0.05	1.85

## Discussion

This study highlights the impact of eyelid mass excision on corneal biomechanics and wavefront parameters. Significant changes were observed in corneal astigmatism, SAs, coma, and LOAs in lesions >5 mm, suggesting an association between a larger lesion size and postoperative changes in selected wavefront parameters. However, overall corneal wavefront parameters and biomechanical properties, including compensated intraocular pressure, CH, and CRF, remained largely unchanged postoperatively.

No significant differences were observed according to the lesion location (middle, medial, or lateral one-third of the eyelid). In subgroup analyses, significant postoperative changes in selected wavefront parameters were observed among lesions measuring >5 mm, particularly in the lower eyelid. These findings should be interpreted cautiously because of the exploratory nature of the subgroup analyses and the relatively small sample size.

In our study, 60% of patients were emmetropic, 30% had myopic astigmatism, and 10% were hypermetropic, findings comparable to those reported by do Nascimento et al., where 58.3% were emmetropic, 33% hyperopic, and 41.7% astigmatic [16].

Although significant postoperative changes were observed in selected wavefront parameters, these findings should be interpreted within the context of the study design. The observed optical changes may reflect alterations in eyelid-induced corneal contour following lesion excision; however, the potential contribution of postoperative ocular surface changes cannot be completely excluded. Furthermore, the absence of significant changes in corneal hysteresis and corneal resistance factor suggests that eyelid surgery may have a greater influence on corneal optical quality than on intrinsic corneal biomechanical properties. These findings represent associations observed in the present cohort and should not be interpreted as establishing a causal relationship.

Ilhan et al. conducted a study involving 29 patients to assess the effects of chalazion surgery on intraocular pressure and corneal properties. They reported a statistically significant reduction in IOPcc (P = 0.020 and P = 0.007) postoperatively, while corneal hysteresis and corneal resistance factor showed no significant postoperative changes (P > 0.05) [17]. In our study, no significant reduction in corneal-compensated intraocular pressure (P = 0.198) or IOPg (P = 0.358) was observed following eyelid surgery. Similar to the findings of Ilhan et al., no significant postoperative changes in corneal hysteresis or corneal resistance factor were noted.

In contrast to previous studies that focused on centrally located upper eyelid chalazia and reported a significant decrease in higher-order aberrations following excision, our study included eyelid masses of varying sizes and locations [18]. Centrally located masses may exert more uniform pressure on the cornea, potentially altering its biomechanics and wavefront. In our cohort, only 20% of chalazia were centrally located, which may explain the lack of significant changes in keratometry and higher-order aberrations following surgery.

Sabermoghaddam et al. reported a reduction in vertical trefoil (0.16 ± 0.05 μm to 0.11 ± 0.06 μm, P = 0.010), horizontal trefoil (0.14 ± 0.04 μm to 0.09 ± 0.05 μm, P = 0.013), and horizontal coma (0.18 ± 0.06 μm to 0.12 ± 0.05 μm, P = 0.021) following chalazion excision [10]. In contrast, our study did not observe significant postoperative changes in total HOAs (P = 0.894), SAs (P = 0.984), coma (P = 0.554), or trefoil (P = 0.415). These differences may be attributed to variations in lesion size and location, as larger or centrally located masses may exert greater mechanical influence on corneal optics.

Park and Lee evaluated the effects of chalazion on corneal SAs and proposed a size threshold for surgical excision [19]. Their study included centrally located upper eyelid chalazia ≥3 mm, dividing participants into two groups (3-5 mm and >5 mm). Using the Galilei analyzer, they observed a significant reduction in total higher-order aberrations and vertical/horizontal trefoil in larger lesions (>5 mm, P < 0.05) and a decrease in astigmatism in both groups (P = 0.040). In contrast, our study did not demonstrate significant changes in astigmatism or higher-order aberrations in smaller (<5 mm) or paracentrally located lesions.

Jin et al. reported that corneal aberrations, including total root-mean-square error, astigmatism, second-order aberrations, and oblique astigmatism, were significantly greater in the upper eyelid chalazion group (P < 0.05), with larger chalazia contributing to more pronounced wavefront aberrations [12]. In contrast, our study did not identify significant differences in astigmatism (P = 0.517), total higher-order aberrations (P = 0.635), coma (P = 0.229), or trefoil (P = 0.496) between upper and lower eyelid chalazia. However, in lesions >5 mm, significant postoperative changes were observed in astigmatism (P = 0.029), SAs (P = 0.008), coma (P = 0.050), and total LOAs (P = 0.049). These variations may partly reflect differences in diagnostic instruments used across studies.

Swaify et al. reported that chalazia involving the lower eyelid produced greater trefoil aberrations compared with upper eyelid chalazia and controls (P = 0.015 and P = 0.001) [11]. They also observed that large chalazia significantly increased trefoil aberrations (P = 0.004 and P = 0.003). In our study, significant changes in astigmatism were observed in lesions larger than 5 mm, suggesting that while chalazia may not always affect refractive error or corneal astigmatism, they can influence higher-order aberrations and visual quality.

Oncul et al. reported a significant reduction in total root-mean-square aberrations following chalazion excision (P = 0.007), while SAs did not change significantly (P = 0.104), findings that are partially consistent with our results [20]. In our study, significant changes in corneal wavefront parameters were observed in lesions ≥5 mm, including astigmatism (P = 0.029), SAs (P = 0.008), coma (P = 0.050), and total LOAs (P = 0.049). However, no significant changes were observed in corneal biomechanical parameters postoperatively.

The present study has several strengths. It was designed as a prospective interventional study with standardized preoperative and postoperative assessment using objective measurements of corneal biomechanics and wavefront aberrations. All surgical procedures were performed by a single surgeon following standardized surgical techniques, thereby minimizing inter-surgeon variability. Patients were evaluated at a uniform postoperative interval, and the study included a heterogeneous spectrum of benign eyelid masses encountered in routine clinical practice, enhancing the clinical applicability of the findings.

This study has certain limitations. First, the sample size was relatively small (n = 20), which may have reduced statistical power and limited the generalizability of the findings. Second, this was a single-center study without a control group, which may limit the external validity of the results. Third, only eyelid masses measuring less than 1 cm were included, and therefore, the findings may not be directly applicable to larger lesions. In addition, multiple statistical comparisons were performed without adjustment for multiplicity; therefore, statistically significant findings, particularly those with borderline P-values, should be interpreted with appropriate caution. Finally, the postoperative follow-up period of six weeks may not have been sufficient to assess long-term corneal biomechanical remodeling following eyelid surgery. Larger multicenter prospective studies with longer follow-up and a broader range of lesion types and sizes are warranted to further validate these findings.

## Conclusions

Excision of eyelid masses may influence corneal optical quality, particularly in lesions measuring ≥5 mm, which demonstrated significant postoperative changes in astigmatism, SAs, coma, and LOAs. However, corneal biomechanical parameters, including CH and CRF, remained largely unchanged following surgery. These findings suggest that postoperative changes in selected wavefront parameters were more frequently observed among lesions measuring ≥5 mm, while no significant differences were observed according to the lesion location. Larger prospective studies are required to further clarify the relationship between eyelid lesions, corneal wavefront changes, and corneal biomechanics following surgical excision.
